# A community health worker and mobile health app intervention to improve adherence to HIV medication among persons with HIV: the CHAMPS study protocol

**DOI:** 10.1186/s12889-023-15616-9

**Published:** 2023-05-24

**Authors:** Olivia R. Wood, Rebecca Schnall, Emma S. Kay, Haomiao Jia, Joseph A. Abua, Tyler K. Nichols, Susan A. Olender, Michael J. Mugavero, D. Scott Batey

**Affiliations:** 1 School of Nursing, Columbia University, 560 West 168th Street, New York, NY 10032 USA; 2 Magic City Research Institute, Birmingham AIDS Outreach, Birmingham, AL USA; 3 Birmingham AIDS Outreach, Birmingham, AL USA; 4 Department of Medicine, Columbia University Irving Medical Center, New York, NY USA; 5 University of Alabama at Birmingham, Birmingham, AL USA; 6 Tulane University, New Orleans, LA USA

**Keywords:** HIV/AIDS, Sexual minority, Community health worker, mHealth, ART adherence

## Abstract

**Background:**

Persons with HIV (PWH) can now achieve a near-normal life expectancy due to antiretroviral therapy (ART). Despite widespread availability of ART in the United States (US), many of the country’s approximate 1.1 million PWH are not achieving viral suppression due to poor ART adherence. Viral suppression rates are particularly low in Alabama (AL, 62%) and New York City (NYC, 67%). There is mixed evidence on the efficacy of community health workers (CHW) and mHealth interventions for improving ART adherence and viral suppression in PWH thus, we sought to combine these interventions and test the efficacy for improving health outcomes in PWH.

**Methods:**

The CHAMPS study is a two-arm randomized controlled trial among 300 PWH with suboptimal primary care appointment adherence (*n* = 150 in AL and 150 in NYC) over the course of 12 months. Participants are randomly assigned to CHAMPS (intervention) or a standard-of-care (control) arm. Participants in the intervention arm are given a CleverCap pill bottle that syncs to the WiseApp to track medication adherence, reminds users to take their medication at a set time, and enables communication with CHW. All participants complete baseline, 6-month, and 12-month follow-up visits where surveys are administered and, CD4 and HIV-1 viral load are obtained through blood draw.

**Discussion:**

Maintaining ART adherence has significant implications in HIV management and transmission. mHealth technologies have been shown to optimize the provision of health services, produce positive changes in health behavior, and significantly improve health outcomes. CHW interventions also provide personal support to PWH. The combination of these strategies may provide the necessary intensity to increase ART adherence and clinic attendance among PWH at highest risk for low engagement. Delivering care remotely enables CHW to contact, assess, and support numerous participants throughout the day, reducing burden on CHW and potentially improving intervention durability for PWH. The adoption of the WiseApp coupled with community health worker sessions in the CHAMPS study has the potential to improve HIV health outcomes, and will add to the growing knowledge of mHealth and CHW efforts to improve PWH medication adherence and viral suppression.

**Trial registration:**

This trial was registered with Clinicaltrials.gov (NCT04562649) on 9/24/20.

## Background

Persons with HIV (PWH) now achieve near-normal life expectancy due to antiretroviral therapy (ART), which has transformed HIV from a terminal diagnosis to a manageable chronic condition [1–4]. However, despite the widespread availability of ART in the United States (US), many of the country’s approximate 1.1 million PWH [5]—in diverse geographic locations—are not fully benefitting from ART due to poor adherence [6]. Suboptimal ART adherence is especially challenging in the US South which accounts for 51% of new domestic cases annually [7]. The Southern HIV epidemic is even more pronounced in the “Deep South” [8] with striking numbers in Alabama (AL) where 62% of PWH experience viral suppression (the ultimate goal of ART) [9]. Similarly, the heavy HIV burden in the US Northeast [10] is in New York City (NYC) which accounts for 82% of all PWH in New York State [11]. Further, in the NYC region, only 67% of PWH achieve sustained, or durable, viral suppression. These suboptimal HIV health outcomes occur within a fragmented healthcare system in the US, [12] further exacerbating the challenges inherent in the lives of underserved, marginalized groups, including many PWH [13]. In response, the US government prioritized the State of AL and four NYC-area counties (study site locations) as five of 55 areas with a substantial burden of HIV in the Ending the HIV Epidemic (EHE) plan for America [14].

The Deep South, a colloquial term comprising nine US states, including AL, had the highest HIV diagnosis rates of any US region during the past decade [7]. Poorer HIV-related health outcomes are seen in AL as compared to other regions in the Deep South, with higher HIV-related mortality rates [15], higher rates of AIDS diagnoses [16] (oftentimes an indicator of late diagnosis or poor disease management [17–19]), and lower rates of engagement in HIV care and viral suppression [20]. While characteristically different from AL, NYC continues to have high HIV diagnosis rates as well [6]. Poorer HIV-health outcomes in the Bronx as compared to other NYC boroughs, have been observed with higher HIV-related mortality rates [21], higher rates of AIDS diagnoses, [6] and lower rates of viral suppression [22]. In addition to geographic variability in HIV health outcomes within our study sites, racial HIV health disparities are also particularly pronounced in the US South and Northeast, specifically AL and NYC. In AL, HIV disproportionally affects racial minority groups, with African-Americans comprising 64% of all PWH and 68% of new diagnoses through the first three quarters of 2022 [23]. HIV also disproportionally affects racial minority groups in NYC, with African-Americans comprising 44% of PWH and 46% of new diagnoses in 2018 [24].

Progression of HIV disease and premature deaths among PWH have been attributed foremost to insufficient engagement in medical care and adherence to HIV treatment regimens [25]. Following diagnosis, rapid access to ART and subsequent sustained ART adherence is central to therapeutic success and is a critical determinant of long-term health outcomes (e.g., viral suppression) in PWH [26–28]. For many chronic diseases, such as diabetes or hypertension, drug regimens remain effective even after treatment is resumed following a period of interruption. In the case of HIV, however, loss of virologic control as a consequence of ART non-adherence may lead to emergence of drug resistance and loss of future treatment options [29–31]. Thus, there is an urgent need to develop and evaluate interventions to enhance ART adherence, and interventions that combine community health workers (CHW) and mHealth technology hold promise for addressing these challenges in the US [32, 33].

Our team conducted two prior studies to support the independent use of CHW and mHealth interventions as described. Firstly, the Birmingham Access to Care (BA2C) study (NCT02525146) was a single-site randomized controlled trial (RCT) funded by AIDS United and conducted between July 2013 and February 2016 in partnership with Birmingham AIDS Outreach (BAO), and the University of Alabama at Birmingham (UAB) 1917 HIV Outpatient Clinic. The study focused on re-engagement in care among PWH who had previously established care but were not engaged at the time of the study. The BA2C intervention was based on a CDC-recognized evidence-based intervention, Anti-Retroviral Treatment and Access to Services (ARTAS), which was the first RCT shown to significantly increase linkage to care among recently diagnosed PWH [34–36]. The ARTAS intervention consists of a brief, strengths-based case management/Motivational Interviewing (MI) approach aimed at facilitating a close, supportive relationship between the community health worker interventionist and participant within up to five sessions. BA2C, which tailored and adapted ARTAS to improve re-engagement of PWH who dropped out of care, provided additional support over a longer period. Participants assigned to the intervention arm received an initial face-to-face meeting with an assigned interventionist and attended at least ten, and up to 12 in-person or telephone sessions over six months during which interventionists worked collaboratively with participants to resolve any barriers to care. Study findings from BA2C showed that participants in both study arms improved across multiple outcomes (re-engagement to care 30 days after baseline; retention in care, ART prescription, and viral suppression at 12 months after baseline), but there was no statistically significant difference between groups. The lack of statistical significance was likely due to a small sample size and a 70% retention rate resultant of challenges that participants had in attending in-person study appointments.

More so, our WiseApp study (NCT03205982) utilized a self-management app for PWH [37] with the goal of being more widely applicable across chronic illness populations who require medications and would benefit from additional self-management strategies. A comprehensive process for the design of the app was guided by the Information Systems Research (ISR) framework and incorporated end-user feedback throughout the design process [38]. The resultant WiseApp was comprised of the following functional components: 1) PWH testimonial videos, 2) push-notification reminders, 3) medication trackers, 4) health surveys, and 5) a “To-Do” list outlining participant tasks for the day, such as medications to take. A key component of the WiseApp is a medication tracker linked to the CleverCap, an electronic pill bottle that sends push-notification reminders to take medication at certain times of day and tracks medication adherence over time, by sending a signal to the app each time the lid of the bottle is opened. WiseApp has the capability to send tailored reminders based on the feedback from the linked devices, such as medication reminders if the pill bottle has not been opened. Preliminary data from this study showed evidence for the success of the intervention in comparison to the control group for improving ART adherence–but not viral suppression–in low-income racial and ethnic minority PWH [39].

### Study objective

Building on our preliminary work, the CHAMPS study aims to enable PWH in the two high priority settings of Birmingham, AL and NYC to self-manage their ART regimens supported by bidirectional communication via mHealth technology and CHW who monitor their ART adherence in real-time through the use of the WiseApp App and provide support in overcoming barriers to HIV care. Specifically, the study is an RCT that assesses the efficacy and sustainability of CHAMPS on viral suppression (primary outcome) and ART adherence (secondary outcome) compared to the standard of care (control group) over 6 and 12 months, as well as to identify mediators and moderators on study outcomes. This paper provides an overview of the CHAMPS protocol.

### Ethics and consent

All study procedures were reviewed and approved by the Western Institutional Review Board. Study participants will provide written informed consent and HIPAA authorization prior to enrollment. Researchers follow institutional policies on data collection and management procedures.

## Methods/design

### Design

The study is a two-arm RCT among 300 PWH (*n* = 150 in AL and 150 in NYC) over 12 months. Participants are randomly assigned to CHAMPS (intervention) or a standard-of-care (control) arm. Comparison of study arms is illustrated in Table 1.

**Table 1:** Comparison of study arms

	**CHAMPS (Intervention)**	**Standard of Care (Control)**
Online survey at baseline, 6-month, and 12-month	X	X
Standard of Care HIV medical care and ancillary services	X	X
Blood Draw	X	X
10 CHW in-person or virtual sessions	X	
WiseApp App and CleverCap pill bottle	X	

#### Intervention arm

The CHAMPS intervention is a six-month intervention guided by our previous work.

#### WiseApp and clevercap pill bottle

The CleverCap pill bottle is an innovative technology that dispenses only the prescribed amount of medication, keeps track of medications dispensed, and communicates wirelessly with the WiseApp. Participants can self-monitor their medications, and their community health worker can track their ART adherence in real time. Participants receive reminders through the WiseApp when they have not taken their medication on time and receive encouraging messages when they take their medication.

#### CHW sessions

CHW administer up to ten individual sessions with participants throughout the course of the intervention. Sessions focus on topics such as re-engagement in care, medication adherence, health literacy, access to support services, and HIV disclosure, as outlined in Table 2. While the first two sessions are conducted in-person, the remaining visits are conducted either in-person or through the chat feature of the WiseApp, depending on participant's preference. Because the CHW sessions can be delivered remotely through the WiseApp, the intervention is able to overcome many common limitations of similar interventions, such as low attendance at in-person sessions due to scheduling conflicts, distance, or cost of travel and childcare. Foundational to intervention sessions is the enhanced personal contact and resultant supportive relationship that is developed between participants and CHW.

**Table 2:** Outline and description of CHW sessions with study participants guided by BA2C and ARTAS content

Title		Sample content for each session
1	Building the Relationship	Introduce the goals of *CHAMPS*
2	Introduction to the WiseApp	Discuss how the App can be used to facilitate communication between CHW and the participant. Review the medication tracking function and how this can be used by CHW and the participant
3	Emphasizing Personal Strengths	To help the participant self-identify personal strengths, abilities, and skills
4	Learning to Make Contact	Assist participant in preparing a list of questions to ask care provider
5	Reminder Call	Call at the agreed upon time; Remind participant of any needed documents and address any potential barriers to care
6	Primary Care Provider Appointment #1	Support participant’s efforts during care provider visit
7	Debriefing Provider Visit with Client #1	Solicit participant’s input on what went well for the participant. Elicit from the participant what was learned from the care visit and what strengths they demonstrated during the care visit
8	Reviewing Progress	Plan for and review the transition process between CHW and study participant
9	Debriefing Provider Visit with Client #2	Solicit participant’s input on what went well for the participant. Elicit from the participant what was learned from the care visit and what strengths they demonstrated during the care visit
10	Completing the work	Review the transition process; transition to standard of care case manager and/or other providers

Prior to working with participants, CHW were trained via Zoom on the intervention, including the content of each session, MI, strengths-based case management, ARTAS, [34] HIV and substance use, and safety in the field. CHW were also trained on the WiseApp to assure successful use of the mHealth technology when working with participants.

### Standard of care (Control Arm)

The control condition includes standard health services offered at each site, including clinical care and referrals to mental health and ancillary services as indicated. Participants in need of linkage to social services are connected to site-specific resources. In summary, standard of care at each site is comprehensive and follows the Department of Health and Human Services HIV guidelines [40].

### Recruitment and eligibility

Participants are eligible to participate if they are 1) Able to speak, read, and write in English or Spanish (NYC site only); 2) aged ≥ 18 years; 3) willing to participate in any assigned arm of the intervention; 4) diagnosed with HIV ≥ 6 months ago; 5) Have an HIV-1 RNA level ≥ 200 copies/mL, or at least one “no-show” visit, in the past 12 months, or report being virally unsuppressed in the past 12 months; 6) own a smartphone; and 7) ability and willingness to provide informed consent for study participation and consent for access to medical records. Participants are not eligible if they meet any exclusion criteria, including: 1) Reside in a nursing home, prison, and/or receiving in-patient psychiatric care at time of enrollment; 2) terminal illness with life expectancy < 6 months; 3) planning to move out of the area in the next 12 months; and/or participating in a study that targets viral suppression for PWH. The study team carefully considered including newly diagnosed participants (diagnosed < 6 months) and chose not to include this subset of PWH because newly diagnosed individuals are often treatment naïve; thus, their adherence behaviors are unknown and may change frequently as they begin their ART regimen.

Participants are recruited using the following strategies (these approaches have been successfully used by our team in past studies [41–45]): Flyers posted at community organizations and clinics where PWH are served, online postings on Craigslist, and pull of medical record data of potentially eligible participants. Consistent with the multi-pronged recruitment approach designed to reduce recruitment bias [46, 47] and to minimize potential recruitment problems, our team carefully monitors our recruitment approaches.

### Sample size calculation

The statistical power was calculated based on the primary outcome of viral suppression (viral load ≤ 200 copies/mL). 300 participants will be enrolled (*n* = 150 in each site) with a 1:1 random assignment to the intervention arm and the control arm (i.e., 75 in each arm per site). All power estimates are based on α = 0.05 and 2-sided tests and the following assumptions: (1) an 80% retention rate at each follow-up assessment for each study arm; (2) a correlation of 0.6 of outcome measure for participants at different time points of assessment; (3) an intra-cluster correlation (ICC) of 0.2 of participants of same study sites; and (4) 75% baseline viral suppression rate (based on preliminary data of the BA2C study). For the total sample (*n* = 300), we calculated power of at least 80% in order to detect a difference of 12% or greater in viral suppression. The 12% difference in viral suppression is equivalent to a small effect size (Cohen’s D of 0.31).

### Randomization

Randomization was achieved through a blocked design utilizing permuted blocks of random sizes in order to achieve a minimally biased assignment of subjects to study arms. The design ensures equal representation of treatment assignment across groups and protects the study team and investigators from easily anticipating treatment allocation [45–48]. Randomization to CHAMPS or standard of care is 1:1. The randomization database is stored on a password protected computer at Columbia University and is accessible to study Principal Investigators to avoid the possibility of the study sites subverting randomization as has been noted in previous studies [49]. Following completion of the informed consent and baseline assessment, participants are randomly assigned to one of two trial arms using sequentially numbered, opaque, sealed envelopes containing the intervention assignment, which the staff member opens at the moment of randomization [50]. Additionally, the HIV literature is limited in interventions among women and racial/ethnic minorities [51]. Because of this, we are dictating enrollment of a minimum of 50% African-American, Hispanic, and Asian participants and 50% female-identifying participants. A minimum of 50% women or transgender women combined across sites will be enrolled, and both sites will stop enrollment of male-identifying participants at 150 to meet this goal.

### Study assessments

Participants are enrolled in-person at both study sites. After enrollment, participants complete study assessments at baseline as well as 6- and 12-month follow-ups via Qualtrics. Participants are required to show a personal ID at all study visits, and study data are securely stored at the primary study site in a limited access database by study ID.

The study outcomes are described in Table 3. The primary outcome of viral suppression is operationalized as viral load ≤ 200 copies/mL at six months. Viral load is the biologic correlate of the ART adherence behavior; thus, to achieve biologic change, there must be change in the adherence behavior. As suggested through the “Undetectable equals Untransmittable,” or “U = U,” public health campaign, [52, 53] PWH who take ART as prescribed and achieve and maintain viral suppression have effectively no risk of sexually transmitting the virus to a serodiscordant partner [52]. Our secondary and related outcomes include ART adherence measured in two ways: 1) an empirically validated, single-item, self-report measure, [54] and 2) electronic pill bottle data, collected via the CleverCap. The electronic pill bottle data provide a less subjective measure as compared to self-report.

**Table 3:** Measures of schedule of events

	**Screening**	**Baseline**	**6 mos**	**12 mos**
Sociodemographic: (e.g., age, race/ethnicity, education, housing)		**X**		
**Primary Outcome Measures**				
Viral Load through a blood draw	**X**		**X**	**X**
**Secondary Outcome Measures**				
ART adherence (SRSI) [54] and the CleverCap (intervention group only)	**X**		**X**	**X**
**Additional Outcome Measures**				
Quality of Life (PROMIS-29) [55]		**X**	**X**	**X**
HIV Symptom Index [56]		**X**	**X**	**X**
Engagement in HIV Care [57]				
**Mediators**				
The HIV Medication Taking Self-Efficacy Sale		**X**	**X**	**X**
Motivation and outcome expectancies of ART adherence are assessed as three separate dimensions: attitudes, norms, and behavioral intentions to adhere to ART medication [58]		**X**	**X**	**X**
Self-regulation skills, which include self-monitoring, goal setting, and enlistment of self-incentives/plans [59]		**X**	**X**	**X**
HIV-regulated Stigma [60]		**X**	**X**	**X**
**Moderators**				
Alcohol, Smoking & Substance Involvement Screening Test (ASSIST) [61]		**X**	**X**	**X**
Depression and Anxiety (Beck Symptom Inventory) [62]		**X**	**X**	**X**

Several mediators are hypothesized to explain the mechanisms through which the intervention is anticipated to improve viral suppression. Hypothesized mediators include self-efficacy, motivation expectancies, self-regulation skills, and HIV-related stigma. The HIV Medication Taking Self-Efficacy Scale is also used to measure ART adherence self-efficacy, or the confidence to take HIV medications in various situations [63]. Motivation and outcome expectancies of ART adherence are assessed as three separate dimensions: attitudes, norms, and behavioral intentions to adhere to ART medication [58]. Self-regulation skills which include self-monitoring, goal-setting, and enlistment of self-incentives/plans, are also assessed. [59] In prior studies of ART adherence, HIV-related stigma and discrimination have been strongly associated with non-adherence; [14, 64] thus, we also measure HIV-related stigma.

Additionally, several moderators are hypothesized to assess the strength of the intervention to yield improvements in viral suppression and will be explored through data analysis. Hypothesized moderators include depression, anxiety, and substance use. Depression and anxiety are measured through the Brief Symptom Inventory, a multi-item scale of mental health in the last seven days that gives a global index and nine primary symptom domains, including depression and anxiety [65]. Alcohol and drug use are assessed with the Alcohol, Smoking & Substance Involvement Screening Test (ASSIST), assessing frequency of use and associated problems for each substance with good to excellent reliability and validity [66].

### Statistical analysis

All multivariate analyses will be preceded by standard descriptive bivariate analyses to describe key variables and relationships among them. These analyses will include means, frequency tables, histograms, and examination of distributions. Our primary outcomes will be a comparison of viral suppression (primary outcome) between CHAMPS and standard of care. We will also compare decrease in viral load (measured in logarithmic scale with base 10) between the two groups. Our secondary and related outcome is ART adherence measured in two ways: 1) a single-item, self-report, empirically validated measure (SRSI) [54] and 2) electronic pill bottle data which we will be collecting in the intervention group since all participants will receive a CleverCap bottle for their medications. All analyses will be based on initial assignment to groups, using the intention-to-treat principle [67, 68].

The primary hypothesis will be tested using a generalized linear mixed model (GLMM) with logit-link function for binary outcomes (i.e., viral suppression) or a linear mixed model (LMM) for continuous outcomes (i.e., viral load measured in logarithmic scale) to account for the non-independence of repeated measurements within individuals [69]. The models will include a random intercept and fixed effects for intervention group, time, and interaction term of group and time which is for testing efficacy of the intervention. The model may include stratification variables, such as study site, age, and sex as covariates. A site by group interaction will also be examined and included in each model (above) if significant at the 0.05 level. All analyses will be tested for goodness-of-fit using the Wald-type test, which shows satisfactory performance for models with fewer (< 5) covariates [70]. A similar GLMM will be used to test sustainability of the intervention at Month 12. For this analysis, we will conduct a non-inferiority test [71] to compare viral suppression rate between Month 12 and Month 6. For primary hypothesis 2, testing for intervention effect on ART adherence measured by SRSI (secondary outcome), we will use a similar LMM as mentioned above. For electronic pill bottle data in the intervention group, we will also examine the trend of electronic pill bottle data over time with a GLMM which will include a first-order autoregressive (AR1) covariance structure [69]. We will also aggregate the electronic pill bottle data at different time points (0, 6, and 12 months) and examine the association between the electronic pill bottle data and ART adherence data measured by SRSI using Spearman nonparametric correlation [54]. Similar GLMMs or LMMs will be used for secondary outcomes, with GLMMs for binary outcomes and LMMs for continuous outcomes. We will measure the number of CHW sessions completed and consider this as a measure of dosage on the intervention in our final analytic model.

## Discussion

Maintaining ART adherence has significant implications in HIV management and onward transmission [72]. Thus, identifying barriers to ART adherence and solutions to mitigate these barriers is vital to the health of PWH, especially those who may struggle to adhere due to economic, social, or other limitations [73]. The combination intervention of the WiseApp and the CHW as described in this paper has the potential to increase ART adherence among our study populations in Birmingham, AL and NYC, as well as nationally. Conducting the CHAMPS study in such disparate study settings will provide critical early information regarding the efficacy of the combination intervention to inform future iterations and dissemination across myriad settings.

The guiding framework of the RCT is the conceptual model of supportive accountability, which is based on the premise that human support increases medication adherence through accountability to a coach—i.e., CHW—who is perceived as trustworthy, knowledgeable, and benevolent [74]. The rapport between participants and CHW is contingent on support and motivation. The role of CHW is based on having an active presence in a participant’s life and fostering culturally competent and respectful communication with participants. [75]. Thus, setting a foundation encourages mutual information sharing, more accessible communication with other healthcare workers (on behalf of CHW), and teamwork. Participant visibility, trust, and monitoring can be gained and encouraged through mobile and in-person communication [76]. In the CHAMPS study, CHW communication includes one-on-one participant education, supportive management, and linkage to quality health services. Thus, the results of the CHAMPS study will be important in determining the role that CHW play in supporting medication adherence among our study population, as well as more broadly. Beyond ART adherence, social support theory suggests that an ongoing alliance between study participants and CHW could protect against depression, substance use, and anxiety [77–79]. Surveys administered in CHAMPS include questions that measure such variables over the course of the study, thus enabling the study team to determine if the presence or absence of CHW impacted self-reported mental health measures over time. The provision of CHW or another individual who can act as support to a participant may be crucial in determining if a participant feels supported and empowered and, ultimately, if they can maintain adherence to prescribed medication.

Utilizing mHealth technologies in CHAMPS may increase ART adherence [80]. For instance, delivering care remotely enables CHW to assess, contact, and support numerous participants throughout the day, amplifying outreach efforts and reducing visit delays due to transportation and time costs [81]. Therefore, mHealth serves as a tool for decreasing healthcare worker burnout while maximizing resources (i.e., educational materials and support services). In turn, through the mHealth component of the CHAMPS intervention, it is hoped that CHW will be able to strengthen ART adherence and viral suppression in PWH. In addition to CHW efficiency, mHealth's timely data monitoring boosts health outcomes by monitoring and addressing the immediate needs of PWH in real-time. Previous research on mHealth interventions has relied on self-reported ART adherence, which may result in overstated or incorrect reports of medication adherence [82]. Thus, the CleverCap App tracking allows CHW to address ART non-compliance promptly and gives them the necessary surveillance tools to coordinate personable treatment plans for PWH. Following the first two in-person sessions, CHW in the CHAMPS study can conduct hybrid visits that cater to participants’ schedules, increasing engagement and participant satisfaction with the intervention. These factors can influence the PWH continuum of high-quality care and the participant’s likelihood of adhering to ART regimens. 

Importantly, the COVID-19 pandemic impacted healthcare approaches worldwide, prioritizing online care and restricting in-person access to healthcare providers, such as CHW [83]. During this time, the use of mHealth technologies as a knowledge dissemination tactic has been shown to positively affect community health behaviors and lifestyle changes [84]. The provision of virtual healthcare not only benefits participants, but also improves CHW performance. CHW reported mHealth to be beneficial for navigating training and staffing issues, as well as avoiding transportation delays [85]. The improvement of online health services can advance the health of PWH and the health system(s) CHW are assisting. The inclusion of CHW and mHealth in the CHAMPS study may result in increased participant engagement in the intervention. However, CHW who deliver care online face the challenge of adopting mHealth technologies, as some CHW may lack knowledge about mHealth technical support and internet connectivity [86]. Despite limitations, the provision of CHW for PWH is a strategic community intervention, and having online access to CHW may increase participant satisfaction, ART adherence, and overall interaction with the intervention. In summary, the CHAMPS study has the potential to add to the growing knowledge of mHealth and CHW efforts to improve PWH medication adherence and viral suppression.

## Data Availability

The datasets generated and/or analysed during the current study are not publicly available due the sensitive health information (HIV status) of the participants) but are available from the corresponding author on reasonable request.

## Abbreviations

PWH: Persons with HIV
ART: Antiretroviral therapy
US: United States
CHW: Community health workers
AL: Alabama
NYC: New York City
EHE: Ending the HIV Epidemic
RCT: Randomized controlled trial
BAO: Birmingham AIDS Outreach
MI: Motivational Interviewing
ISR: Information Systems Research
ASSIST: Alcohol, Smoking & Substance Involvement Screening Test
GLMM: Generalized linear mixed model
LMM: Linear mixed model
